# Integrating a Statistical Topic Model and a Diagnostic Classification Model for Analyzing Items in a Mixed Format Assessment

**DOI:** 10.3389/fpsyg.2020.579199

**Published:** 2021-02-09

**Authors:** Hye-Jeong Choi, Seohyun Kim, Allan S. Cohen, Jonathan Templin, Yasemin Copur-Gencturk

**Affiliations:** ^1^Georgia Center for Assessment, Department of Educational Psychology, University of Georgia, Athens, GA, United States; ^2^Department of Psychology, University of Virginia, Charlottesville, VA, United States; ^3^Department of Psychological and Quantitative Foundations, University of Iowa, Iowa City, IA, United States; ^4^Rossier School of Education, University of Southern California, Los Angeles, CA, United States

**Keywords:** text analysis, mixed format test, diagnostic classification model, structural topic model, statistical topic models

## Abstract

Selected response items and constructed response (CR) items are often found in the same test. Conventional psychometric models for these two types of items typically focus on using the scores for correctness of the responses. Recent research suggests, however, that more information may be available from the CR items than just scores for correctness. In this study, we describe an approach in which a statistical topic model along with a diagnostic classification model (DCM) was applied to a mixed item format formative test of English and Language Arts. The DCM was used to estimate students’ mastery status of reading skills. These mastery statuses were then included in a topic model as covariates to predict students’ use of each of the latent topics in their written answers to a CR item. This approach enabled investigation of the effects of mastery status of reading skills on writing patterns. Results indicated that one of the skills, Integration of Knowledge and Ideas, helped detect and explain students’ writing patterns with respect to students’ use of individual topics.

## Introduction

Selected response (SR; e.g., multiple choice or true–false) items and constructed response (CR; e.g., short answer, long answer essay, or performance) items are often found in the same test. An important benefit of SR items is their efficiency in being scored quickly with minimal potential for raters’ bias. CR items, on the other hand, have been shown to be appropriate for assessing certain types of higher order knowledge, as this type of item can be used to require students to construct their answers and frequently show their reasoning in their answers (Brookhart, 2010).

While SR and CR items are used together, existing psychometric approaches do not benefit from both data sources efficiently. Most psychometric models, including item response theory models and diagnostic classification models (DCMs), have been developed for focusing on item scores, i.e., correctness of the responses. This is true for CR items as well. The partial credit model (Masters, 1982) and the general diagnostic model (von Davier, 2008), for example, can be used for CR items, but these models only focus on item scores and do not directly include analysis of students’ constructed responses, when estimating model parameters. As a result, any additional information contained in the text of students’ answers is ignored.

Statistical topic models (Blei, 2012), on the other hand, are designed to detect the latent thematic structure in the textual data. In education, topic models have recently used. For example, Daenekindt and Huisman (2020) used a topic model to investigate trends in research topics in higher education by analyzing journal abstracts. Moretti et al. (2015) explored the use of different topics on teacher evaluation policy by examining research articles found on the internet. Kim et al. (2017) investigated growth and change in use of academic vocabulary as a result of an instructional intervention, and Duong et al. (2019) found that students’ differential use of topics in their CR answers reflected differences in students’ reasoning associated with differences in the instructional training of their teachers.

In this study, we present an approach in which results from a DCM were used in a topic model as covariates to understand the relationship between students’ mastery status of reading skills and the latent thematic structure in students’ writing to answer to a CR item. Specifically, a log-linear cognitive diagnostic model (LCDM; Henson et al., 2009) was used as a DCM and a structural topic model (STM; Roberts et al., 2013) was used as a topic model. This combined use of the two models enabled direct investigation of the relationships between mastery of reading skills and use of latent topics. In the next section, we describe the LCDM and the STM.

## Log-Linear Cognitive Diagnosis Model

Diagnostic classification models (Rupp et al., 2010) are probabilistic models developed to obtain information regarding students’ mastery status on a set of pre-determined skills. DCMs predict response patterns for individual mastery profiles based on the attribute structure given in the Q-matrix for a test. In this way, the DCM provides a deterministic confirmatory framework for the assessment. The DCM also include the capability of accounting for uncertainties in examinees’ behavior on a test, such as guessing or slipping. Several models have been proposed by imposing different conditions for determining the probability of answering the item correctly and handling these kinds of sources of uncertainty.

As a general frame of reference for a DCM, in the LCDM, the probability of getting a correct answer is modeled as a function of item (*j*) parameters and the mastery status of the individual (*i*) given the Q-matrix as follows (Henson et al., 2009):


$$P\left(Y_{i\,j}=1|\alpha_{i},q_{j}\right)=\frac{\text{exp}\,\left[\lambda_{j\,0}+\lambda_{\text{j}}^{\text{T}}\,h\,\left(\alpha_{i},q_{j}\right)\right]}{1+\text{exp}\,\left[\lambda_{j\,0}+\lambda_{j}^{T}\,h\,\left(\alpha_{i},q_{j}\right)\right]},$$

where λ_*j*0_ indicates the intercept, λ_*j*_ represents a vector of coefficients indicating effects of the mastery of attributes on the response for item *j*, and *h*(α_*i*_,*q*_*j*_) is a vector of linear combinations of the α_*i*_ and *q*_*j*_, which specifies an effect structure of the model. *h*(α_*i*_,*q*_*j*_) can include main effect of each attribute, two-way interactions, three-way interactions, etc., depending on how many attributes there exist in the test. For instance, if the effect structure includes only main effects and two-way interactions, the model can be represented as


$$\lambda_{j}^{T}\,h\,\left(\alpha_{i},q_{j}\right)=\sum_{s=1}^{S}\lambda_{j\,s}\,\left(\alpha_{s}\,q_{j\,s}\right)+\sum_{s=1}^{S}\sum_{u>s}^{S}\lambda_{j\,s\,u}\,\left(\alpha_{s}\,\alpha_{u}\,q_{j\,s}\,q_{j\,u}\right)$$

where λ_*js*_ represents the main effects of attribute *s* on item *j* and λ_*jsu*_ represents the two-way interaction effects between the combination of attributes *s* and *u* on item *j*. As indicated earlier, this can be extended to three-way or more interaction terms, if needed. Due to the flexibility of this effect structure, the LCDM provides a general framework for DCMs. Further, one can investigate whether the relationship among attributes is compensatory or non-compensatory. For example, using a significance test for λ_*jsu*_ without predetermining the magnitude of the relationship of the two attributes *s* and *u* on item *j*, the relationship between attributes *s* and *u* on item *j* can be tested.

## Structural Topic Model

Topic models are statistical models designed to extract the latent topic structure in a collection of documents (Blei et al., 2003; Griffiths and Steyvers, 2004). Latent Dirichlet allocation (LDA; Blei et al., 2003) is one of the simplest topic models. It assumes that each document in a corpus is a mixture of topics, and each topic is assumed to have a multinomial distribution over a fixed vocabulary of words. A topic is defined as a mixture over words, where each word has a separate probability of belonging to each topic in the model and each document is assumed to consist of a mixture of topics. In LDA, the topics are latent variables to be inferred from the words in a corpus which are the observed variables. In LDA, the order of the words and the grammatical role of the words in the text are ignored. This is called the “bag of words” assumption (Blei et al., 2003).

Roberts et al. (2013) proposed the STM as an extension of the LDA in which a document-level covariate structure can be included to help detect the latent topics in the corpus of textual data. In the STM, one or more covariates can be added to predict the topic proportions or the word probabilities, or both. In the current study, we focused on the use of covariates for predicting topic proportions. To this end, the generative process for estimating topic proportions with an STM is defined to include a covariate structure for the topic proportions for the document (θ) as follows (Roberts et al., 2013):

– For each document, *d*:
  о Draw the topic proportions for the document (θ_*d*_)∼LogisticNormal(μ,Σ)

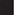

μ_*d*,*k*_ = *X_d_*γ_*k*_

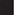



$\gamma_{k}∼N\,\left(0,\sigma_{k}^{2}\right)$

– For each word in the document, [*n* ∈ (1,⋯,*N_d_*)]
  о Draw word’s topic assignment (*z*_*d*,*n*_)∼Multinomial (θ_*d*_)
  о Conditioning on the topic chosen, draw an observed word from that topic (*w*_*d*,*n*_)∼Multinomial (β_*k* = *z*_*d*,*n*__)

where *X*,γ,and Σ are covariates, coefficients, and the covariance matrix, respectively. The coefficients for topic *k* (γ_*k*_) follow normal distributions (mean = 0 and variance = $\sigma_{k}^{2}$). θ_*d*_ denotes a vector for topic proportion for a document, β_*k* = *z*_*d*,*n*__ denotes a vector for word probabilities, and *d* denotes a document that is a sequence of N words (*w*_*d*,*n*_). The inclusion of one or more covariates allows the model to borrow strength from documents with similar covariate values for estimating the document proportion (Roberts et al., 2013). In the current study, we investigated the relationship between students’ reading ability and students’ writing ability by using an STM in which students’ mastery status of reading skills was used as covariates to help explain the use of topics in writing.

**FIGURE 1:**
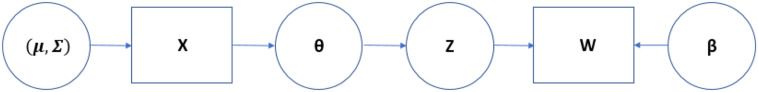
Schematic presentation of structural topic model for this study. Note. The squares indicate the observed variables [i.e., students’ mastery status of reading skills (X) and words in the corpus of students’ writing (W)]; the circles indicate the parameters in the model.

For the current study, the model was set to run for a maximum of 500 EM iterations and convergence was monitored by setting convergence tolerance 0.00001. We used the default options for priors for γand Σ. Figure 1 depicts the model used in the current study.

## Reading and Writing Assessment

Integrated assessments have been used in assessing English language proficiency to enhance the authenticity and validity of assessment (Read, 1990; Feak and Dobson, 1996; Weigle, 2004; Plakans, 2008; Weigle and Parker, 2012). In a typical integrated assessment, students read one or more passages and use the information from the passages as source material to respond to the item. Some borrowing of material is considered appropriate (e.g., used as source material for the answer) but simply copying is not considered appropriate (Weigle and Parker, 2012).

Reading interventions have been shown to help improve students’ writing performance (Graham et al., 2018). Reading and writing skills, although connected, are cognitively separate (Fitzgerald and Shanahan, 2000; Deane et al., 2008; Schoonen, 2019). In this study, the STM topic model along with the LDCM was used to investigate the relationships between reading attributes and writing ability.

## Materials and Methods

### Data and the Q-Matrix

The data consisted of responses of 2,323 Grade 8 students’ responses to the argumentative genre of an English and Language Arts (ELA) test. The test was designed to provide formative information on how well students understood concepts and could demonstrate their knowledge in reading and writing.

#### Skills Measured

The test consisted of five items: three multiple choice items, one short answer (SA) item, and one extended response (ER) item to measure reading and writing ability. Two scores were assigned for the ER item. A confirmatory factor analysis supported this two-factor model: the multiple choice and SA items formed one factor, reading ability, and the two scores for the ER item measured the other factor, writing ability. A non-linear internal consistency estimate (Green and Yang, 2009; Kim et al., 2020) for this two-factor assessment was 0.83, suggesting acceptable reliability (Kline, 2000, p.13).

The multiple choice and SA items were designed to measure three skills: identifying key ideas (*Idea*), identifying the structure of a text (*Structure*), and integrating knowledge of ideas (*Integration).* These three skills were used to create the entries in the Q-matrix shown in Table 1. Three items required a single attribute to answer and one item required two attributes to answer. For the item designed for two attributes, the main effect of each attribute and two-way interactions between these two attributes were identified in the effect structure in the LCDM. *Mplus version 7.4* (Muthén and Muthén, 1998–2017) was used to estimate the LCDM.

**TABLE 1:** Q-Matrix of three reading skills for the multiple-choice and short response items.

Item	Idea	Structure	Integration
Multiple-choice item 1	x		
Multiple-choice item 2		x	
Multiple-choice item 3	x		
Short answer item 4	x		X

To measure writing ability, the ER item consisted of two passages: one passage was about environmental facts and the other was about economic facts. Students were instructed to write an argumentative essay indicating whether their congressional representative should allow the protected forest to be developed into commercial timberland and to support their argument with information from each of the passages. The rubric based score of this item ranged 0–7 points. Partial credit was awarded if part of the response was correct (See Appendix A for the rubric). In the current study, students’ written responses to this item were used to estimate the latent topic structure using the STM as described in more detail in the next section.

### Fitting the Topic Model

The STM topic model was used to identify latent topics in students’ written responses to the ER item and investigate the relationship between reading and writing ability. The first step in applying any topic model is to preprocess the text. This is done to help the estimation process and improve interpretability of the resulting model (Schofield et al., 2017). Preprocessing consists of (1) removing stopwords and (2) stemming words. Stop words are high-frequency but low-information words such as *a, the, that, it, be* (*am, are, is, were, have been, etc.*), *but, or, etc.* Stemming consists of converting words to their root form. For instance, all verbs were converted to the present tense, plural forms were converted to singular form, words that have similar morphology (e.g., do, doing, and done) were converted to a root form such as do, and typographical errors were corrected.

After stemming words and removing stopwords, words with a frequency of less than 10 and documents with less than 15 words were excluded. In addition, documents with a score of 0 were excluded as this indicated the responses were not scorable. As shown in Appendix A, reasons for non-scorability included being blank, simply copying from the passages, answers were written in a language other than English, and answers were too limited, off topic or generally non-responsive to the prompt. The final data set included 2,108 students’ responses with a total of words 270,405 in the corpus. The number of unique words was 891 and the average answer length was 128.3 words (SD = 76.4 words).

The next step was to determine how many latent topics appeared in the data. This is an exploratory analysis. That is, we estimated STM models with from 2 to 20 topics as candidate models. For the STM, students’ mastery statuses on each attribute were included as a set of document-related covariates for predicting the use of topics. There is no single best method for determining the best fitting topic model. Roberts et al. (2014) suggested use of semantic coherence (Mimno et al., 2011) and exclusivity (Bischof and Airoldi, 2012). These two measures are complementary. These indices were used in this study to inform the selection of the best fitting topic model. In addition, the cosine similarity (Cao et al., 2009) between topics was estimated. The lower cosine similarity indicates better fit as this indicates topics are distinct each other. The R package *stm* (Roberts et al., 2019) was used to estimate the STM.

## Results

### Students’ Reading Skill Profiles

For item 4, as no significant interaction effect for attributes 1 and 3, the interaction term was dropped from the effect structure in the final LCDM model. Table 2 presents item parameter estimates for the final model. All main effects were significant at *p* < 0.01. Intercepts for items 1 and 3 were significant (*p* < 0.01), but the intercepts for items 2 and 4 were not. Table 3 presents students’ mastery profiles of the reading skills, the marginal proportions, and reliabilities for each of the skills. Skill reliabilities were relatively low, reflecting the small number of items measuring each skill. The correlation between *Idea* and *Structure* was 0.86, the correlation between *Idea* and *Integration* was 0.67, and the correlation between *Structure* and *Integration* was 0.57. These indicated substantial relationships between skills. Eight different mastery profiles are possible for the three skills in the Q-matrix. Results in Table 3, however, indicate that only four of the eight profiles were detected. These included students who had mastered none of three skills (0,0,0), students who had mastered only *Integration* (0,0,1), students who had mastered *Idea* and *Integration* (1,0,1), and students who had mastered all three skills (1,1,1). Students’ mastery statuses for each attribute obtained by this analysis were included in the STM as covariates to predict the use of topics.

**TABLE 2:** Item parameter estimates for the log-linear cognitive diagnostic model for students’ reading skills.

Item	Intercept	Main effect		
		
		Key idea	Craft and structure	Integration
Multiple-choice item 1	−0.613	1.557	–	–
Multiple-choice item 2	*	–	3.370	–
Multiple-choice item 3	0.434	1.967	–	–
Short answer item 4	*	6.004	–	0.924

*indicates no significance with a significance level of 0.01 and – indicates not applicable given the item.

**TABLE 3:** Students’ mastery status of reading skills and reliability of each skill.

Profile*	Key ideas	Craft and structure	Integration of knowledge and ideas	Count (%)
1 (000)	0	0	0	323 (13.90)
2 (001)	0	0	1	296 (12.74)
3 (010)	0	1	0	0 (0.00)
4 (011)	0	1	1	0 (0.00)
5 (100)	1	0	0	0 (0.00)
6 (101)	1	0	1	146 (6.28)
7 (110)	1	1	0	0 (0.00)
8 (111)	1	1	1	1,558 (67.07)
Marginal proportion**	66%	59%	51%	2,323 (100.00)
Skill reliability	0.69	0.62	0.51	

*0 indicates being classified non-mastery and 1 indicates being classified mastery. **Marginal proportion of students who have mastered each skill.

### Selection of the Topic Model and Interpretation of Topics

**FIGURE 2:**
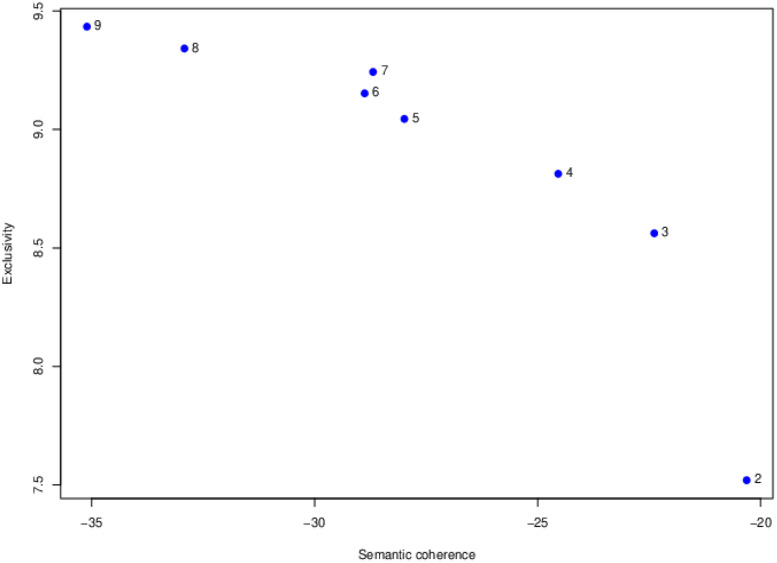
Comparisons semantic coherence and exclusivity among 2- to 9-topic structural topic models.

To detect the number of topics, STM models with from 2 to 20 topics were fit to the data as an exploratory analysis. As described in Methods section, semantic coherence, exclusivity, and cosine similarity were used to determine the number of topics. Figure 2 presents the results of semantic coherence and exclusivity for each of the model with from two to nine topics. The horizontal axis is semantic coherence and the vertical axis is exclusivity. Models in the upper right corner would be models that are higher in both semantic coherence and exclusivity. The best models based on these two indices would be the three- and four-topic models. Cosine similarity results suggested the four-topic model was a better fit than the three-topic model. Based on these results, the four-topic model was selected as the best-fit model.

One way to help interpret and characterize each topic in the model is to examine (1) written responses of students who were the highest probability users of each topic and (2) the highest probability words for each topic. The 15 highest probability words in each topic for the four-topic STM are listed in Table 4. The answer of the student who was the most frequent user of words from each topic is presented below. **The** **bold and underlined words are the highest frequency words for** **the given topics**.

**TABLE 4:** The 15 high frequent words in each topic detected from the 4-topic STM.

Topic 1	Topic 2	Topic 3	Topic 4

Integrative borrowing	Everyday language	Copying from passage	Copying from stem
Pollution	Tree	Georgia	Forest
Paper	Down	Timber	Protect
Mill	For	Acre	Timber
Industry	Cut	More	Should
Coastal	If	Forest	Commercial
Plain	Animal	Coastal	Animal
Water	Make	Plain	Plant
Georgia	Can	For	Species
Passage	More	Pine	Representative
Fish	Because	Commercial	Allow
Cause	People	Industry	Because
Environment	Need	Grow	Develop
Due	Go	Year	Congress
Provide	Get	Passage	Destroy
Forest	Land	Land	Live

In the first topic, the highest frequency words were *pollution*, *paper*, *mill*, *industry*, *coastal*, and *water* (*Pollution* was used as a stemming word for *pollution*, *polluter*, and *pollutant*). These words come from the prompt (i.e., either the two passages in the prompt or the stem of the SR items). Students had been instructed to use information from the passages to support their arguments. This topic was labeled *Integrative Borrowing* as it reflected this use of the terms in the prompt. The following is the answer of the student who was the most frequent user of words from this topic.

(Integrative Borrowing) **Paper mills** are having a negative effect. Passage A says “**Paper mills** are the third largest **polluters** in the United States., releasing **pollutants** into the air, **water**, and soil.” Passage A also say that “many **paper mills** are working to reduce the amount of **pollutants** they produce today.” But they are letting it out, and it also effecting in passage B it says that “the fishing **industry** decreases due to **pollution** caused by **paper mills**.” That why I think **paper mills** are having a negative effect.

The highest frequency words in the second topic were *tree*, *animal*, *cut*, and *people*. These words reflect use of everyday language but not directly related to the question. This topic was labeled as *Everyday Language*. The following is the answer of a student who was a high frequency user of words from topic 2.

(Everyday Language) No because they are killing all the plants and taking the **animals** homes away so how would you feel if someone just took your house away and built something else and just put your family out on the street with nowhere to go, that’s how the **animals** feel. Your destroying our plants life to that we need the plants and **animals** were there first and they really don’t have any other home to go to besides a zoo why do that when they can just be free without the **people** harming them.

The highest frequency words in the third and fourth topics were both borrowed directly from either the stem or the passages. The words in the third topic were copied from the passages (*Georgia*, *timber*, *acre*, *coastal*, *plain*, and *pine*). (*Timber* was used as a stemming word for *timber*, *timberland*, and *timberwood*). The words in the fourth topic were copied from the stem (*forest*, *protect*, *timber*, *should*, *commercial*, *representative*, *allow*, and *congress*). The followings are answers of students who were the highest frequency users of words from the third and the fourth topics, respectively. Characteristic of users of topics 3 and 4 is that these words were simply copied from the passage or stem without any clear effort to integrate the words into the argument.

(Copying from Passages) I think that the small protected forest should not be developed into commercial **timberland** because you don’t have a lot of land. The text states in passage B that “Sixty percent of **Georgia’s coastal** plain is covered in forest. The forest is one of the most diverse ecosystems in America and includes forest, grassland, sandhill, marsh, swamp, and **coastal** habitats. Several varieties of pine and oak are the most common trees. The growth of the ground under the long leaf **pine** forest contains 150–300 plant species per **acre**, more birds than any other **Georgia** forest type, and 60% of the amphibian and reptile species found in the Southeast. The **Georgia** state reptile, the gopher tortoise, lives in **pine** forest habitats and is a key species in the ecosystem. Though once an endangered species, the American alligator is now very common, numbering an estimated 2 million in the Southeast.” This shows that the forest has already been occupied by one of the most diverse ecosystems in America and includes many plants and many amphibian and reptiles. In conclusion this is why I feel like the small protected forest should not be developed into commercial **timberland**.
(Copied from Stem) The **representative should** not **allow** the **protected forest** to be developed into the **commercial timberland**. They **shouldn’t** because, in passage B it states that the soil isn’t suitable for any kind of **forest**. The **timberland** is worth an average of $97 a year because the land isn’t suitable for the tree’s and soil. That is why you **shouldn’t** allow them to put the **protected forest** in the **timberland**.

**FIGURE 3:**
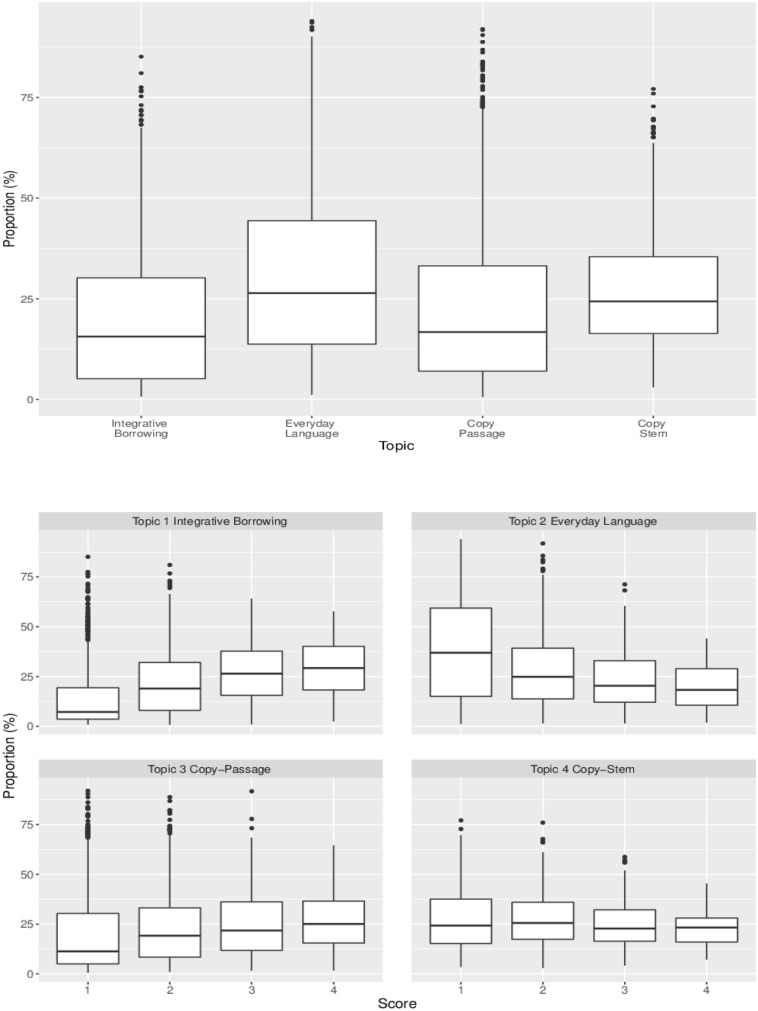
Boxplot for topic proportion distribution. The plot on the upper panel presents the distributions of marginal topic proportions. The plots on the lower two panels present score distributions for each topic. For the plot on the lower panel, the X-axis indicates each score point and the Y-axis indicates the proportions of use of each topic. The whiskers on the boxes indicate variability outside the upper and lower quartiles and the horizontal lines in the boxes indicate the mean usage of the topic for the given score point.

Figure 3 presents box plots of students’ use of individual topics. The plot on the upper panel indicates that overall, students used 20, 31, 22, and 27% of Topics 1, 2, 3, and 4, respectively. The plots on the lower two panels show the rubric based score distribution for each topic. There are two distinct patterns in the Figure 3: (1) students who used more *Integrative Borrowing* in their answers tended to have higher scores and (2) students who used more *Everyday Language* in their answers tended to have lower scores.

### What Is the Relationship Between Students’ Mastery Status of Reading Skills and the Use of the Latent Topics in Writing?

Table 5 presents results for the effects of students’ mastery status of reading skills on their use of each of the four topics in the STM. The values in Table 5 indicate the coefficients for the intercept and for each of the three skills estimated from the DCM. The intercept can be interpreted as the expected use of the topic when students do not master any skills at all, and other coefficients can be interpreted as the expected use of the topic when students master individual skills.

**TABLE 5:** Results of STM for predicting the use of topics by mastery status of reading skills.

	Estimate	SE	*t*-test	Pr(> | t|)

Topic 1: Integrative borrowing				
(Intercept)	0.11	0.012	9.01	0.00
Key ideas	0.03	0.020	1.32	0.19
Craft and structure	0.02	0.017	0.98	0.33
Integration of knowledge and ideas	0.07	0.018	3.77	0.00

**Topic 2: Everyday language**				

(Intercept)	0.49	0.015	32.54	0.00
Key ideas	–0.04	0.023	–1.73	0.08
Craft and structure	–0.03	0.020	–1.45	0.15
Integration of knowledge and ideas	–0.15	0.020	–7.27	0.00

**Topic 3: Copying from passage**				

(Intercept)	0.16	0.014	11.76	0.00
Key ideas	0.02	0.023	0.90	0.37
Craft and structure	0.01	0.020	0.35	0.73
Integration of knowledge and ideas	0.05	0.019	2.60	0.01

**Topic 4: Copying from stem**				

(Intercept)	0.24	0.011	21.77	0.00
Key ideas	–0.01	0.018	–0.42	0.67
Craft and structure	0.01	0.015	0.34	0.74
Integration of knowledge and ideas	0.03	0.015	2.03	0.04

The results indicate mastery status of either *Key Ideas* or *Craft and Structure* did not have a significant impact on students’ use of the topic. *Integration of Knowledge and Ideas* (*Integration*) was the only skill that had a significant effect on the use of each topic at *p* < 0.05. As seen in Figure 3, *Integrative Borrowing* and *Everyday Language* tended to be related to the rubric based score. The results in Table 5 show similar patterns. This suggests that when students master the *Integration*, their probability of using the integrative borrowing topic increases by 0.07, their probability of using the copying from passage topic increases by 0.05, their probability of using the copying from stem topic increases by 0.05, but their probability of using the everyday language topic decreases by 0.15.

## Conclusion

In this study, an approach was described a topic model to obtain the latent thematic structure in students’ written answers to an ER item. In the topic model, results from a DCM applied to the item scores (i.e., the correctness of students’ answers) were included as covariates to predict students’ use of the topics. Although three skills were identified in the Q-matrix, only four of the eight possible mastery profiles were present in the data. The four-topic STM was found to be the best fit to the textual data from the students’ answers to the test questions along with students’ reading skills as covariates. The results showed that mastery status of *Integration of Knowledge and Ideas* was the pivotal skill for the use of each of the four topics. That is, as students mastered *Integration of Knowledge and Ideas*, they tended to use more of the *Integrative Borrowing* topic in their writing and less of the *Everyday Language* topic. CR or ER items are often used to assess higher-order thinking skills. Rubric-based scores provide useful information regarding students’ knowledge status with respect to the objectives being measured on the test. There is also information about students’ thinking and reasoning as reflected in their answers, however, that can be missed by the rubric-based scores alone (Cardozo-Gaibisso et al., 2020). For example, each topic could represent a set of possible misconceptions (Shin et al., 2019) or writing style.

The assessment used in this study was a formative assessment and was not specifically designed to fit a DCM model. Due to the small number of items in the assessment, the skill reliabilities were relatively low, which is a possible limitation of this study. Even with this limitation, however, results demonstrate that combining results from a DCM with a topic model enables the possibility of investigating the relationship between the knowledge as measured by the multiple choice items and cognitive skills used in answering to the CR items. Topic modeling is relatively new in educational research, but it has been found to provide a useful set of methodological tools for extracting this added information in the text of answers to CR items.

Some of current techniques developed in natural language processing or machine learning may not be applicable for the text in education as the text in education may have different characteristics from the text in social networks or publications. Further studies would be helpful to address important issues in this area, such as what could be the effects of stemming methods on latent topic structure or what methods could be used for selecting the best fitting topic model.

## Data Availability Statement

The raw data supporting the conclusions of this article will be made available by the authors, without undue reservation.

## Ethics Statement

The studies involving human participants were reviewed and approved by Human Subjects Office, University of Georgia. Written informed consent from the participants’ legal guardian/next of kin was not required to participate in this study in accordance with the national legislation and the institutional requirements.

## Author Contributions

All authors listed have made a substantial, direct and intellectual contribution to the work, and approved it for publication.

## Conflict of Interest

The authors declare that the research was conducted in the absence of any commercial or financial relationships that could be construed as a potential conflict of interest.

## Appendix

### Appendix A: Rubric for the extended response item

**Appendix A1:** The rubric has two traits: Idea Development, Organization, and Coherence; and Language Usage and Conventions. The scale for Idea Development, Organization, and Coherence ranges from 0 to 4, and the scale for Language Usage and Conventions ranges from 0 to 3.

**Trait 1: Idea development, organization, and coherence.**
**If the student scored 4 points…**
• Effectively introduced a claim or argument.
• Effectively organized the reasons, using logical reasons and evidence.
• Provided clear, relevant reasons/evidence to support the opinion.
• Acknowledged and developed counter-claims, as appropriate.
• Used linking words and phrases effectively to connect opinions and reasons.
• Maintained a formal style appropriate for the task.
• Provided a strong concluding statement or section.
**If the student scored 3 points…**
• Introduced a claim or argument.
• Included an organizational structure that supported the reasons and evidence.
• Provided reasons, facts, and evidence to develop the claim.
• Attempted to introduce a counter-claim, as appropriate.
• Used some linking words to connect opinions and reasons.
• Used a formal style fairly consistently appropriate for the task.
• Provided a concluding statement or section that follows the argument.
**If the student scored 2 points…**
• Attempted to introduce an opinion or a claim.
• Attempted to provide some organization, but structure sometimes impeded the reader.
• Attempted to provide reasons and facts that sometimes support the opinion, but the reasoning is unclear.
• Made no or little attempt to introduce a counter-claim.
• Used few linking words to connect opinions and reasons.
• Used a formal style inconsistently or the style was inappropriate for the task.
• Provided a weak concluding statement or section that does not support the argument.
**If the student scored 1 point…**
• The student did not include a claim or claims, or the claim must be inferred.
• The organizational structure was not evident, not appropriate, or was formulaic.
• There may not have been sufficient support for the claim (if stated).
• The student made no attempt to introduce a counter-claim.
• Very few, if any, linking words and phrases were used.
• Used an informal style not appropriate for the task.
• There was no conclusion, or the conclusion was not related to the essay.
**If the student scored 0 points…**
• The response was blank, copied, or too brief to score.
• The response was illegible, incomprehensible, or was written in another language.
• The response was off topic, off task, or was offensive.
**Trait 2: Language usage and conventions.**
**If the student scored 3 points…**
• There was a variety of sentence types for meaning and interest, and sentences were clear and complete.
• Conventions and language were used appropriately.
• Errors in usage and conventions were infrequent and did not interfere with the meaning of the response.
**If the student scored 2 points…**
• There was some variety of sentence types, and most were complete.
• Demonstrated some knowledge of conventions and language.
• Minor errors in usage did not significantly interfere with the meaning of the response.
• If the student scored 1 point…
• There were fragments, run-ons, and other sentence structure errors.
• Conventions and language were not appropriate.
• Frequent errors in usage interfered with the meaning of the response.
**If the student scored 0 points…**
• The response was blank, copied, or too brief to score.
• The response was illegible, incomprehensible, or was written in another language.
• The response was off topic, off task, or was offensive.
