# Corrigendum: Integrating a Statistical Topic Model and a Diagnostic Classification Model for Analyzing Items in a Mixed Format Assessment

**DOI:** 10.3389/fpsyg.2021.769429

**Published:** 2021-10-11

**Authors:** Hye-Jeong Choi, Seohyun Kim, Allan S. Cohen, Jonathan Templin, Yasemin Copur-Gencturk

**Affiliations:** ^1^Georgia Center for Assessment, Department of Educational Psychology, University of Georgia, Athens, GA, United States; ^2^Department of Psychology, University of Virginia, Charlottesville, VA, United States; ^3^Department of Psychological and Quantitative Foundations, University of Iowa, Iowa City, IA, United States; ^4^Rossier School of Education, University of Southern California, Los Angeles, CA, United States

**Keywords:** text analysis, mixed format test, diagnostic classification model, structural topic model, statistical topic models

In the original publication, an author's name was incorrectly spelled as ‘H.-J. Choi’. The correct spelling is ‘Hye-Jeong Choi’. The authors apologize for this error and state that this does not change the scientific conclusions of the article in any way. The original article has been updated.

## Publisher's Note

All claims expressed in this article are solely those of the authors and do not necessarily represent those of their affiliated organizations, or those of the publisher, the editors and the reviewers. Any product that may be evaluated in this article, or claim that may be made by its manufacturer, is not guaranteed or endorsed by the publisher.

